# BCAT1 decreases the sensitivity of cancer cells to cisplatin by regulating mTOR-mediated autophagy *via* branched-chain amino acid metabolism

**DOI:** 10.1038/s41419-021-03456-7

**Published:** 2021-02-10

**Authors:** Lifang Luo, Wenjing Sun, Weijian Zhu, Shuhan Li, Wenqi Zhang, Xiaohui Xu, Daoquan Fang, Tan Hooi Min Grahn, Lei Jiang, Yihu Zheng

**Affiliations:** 1 Central Laboratory, the First Affiliated Hospital of Wenzhou Medical University, Wenzhou, 325000 China; 2 Division of Molecular Medicine and Gene Therapy, Lund Stem Cell Center, Lund University Hospital, Lund, 22184 Sweden; 3 Department of General Surgery, the First Affiliated Hospital of Wenzhou Medical University, Wenzhou, 325000 China

**Keywords:** Cancer, Cell biology

## Abstract

Cisplatin is one of the most effective chemotherapy drugs and is widely used in the treatment of cancer, including hepatocellular carcinoma (HCC) and cervical cancer, but its therapeutic benefit is limited by the development of resistance. Our previous studies demonstrated that BCAT1 promoted cell proliferation and decreased cisplatin sensitivity in HCC cells. However, the exact role and mechanism of how BCAT1 is involved in cisplatin cytotoxicity remain undefined. In this study, we revealed that cisplatin triggered autophagy in cancer cells, with an increase in BCAT1 expression. The cisplatin-induced up-regulation of BCAT1 decreased the cisplatin sensitivity by regulating autophagy through the mTOR signaling pathway. In addition, branched-chain amino acids or leucine treatment inhibited cisplatin- or BCAT1-mediated autophagy and increased cisplatin sensitivity by activating mTOR signaling in cancer cells. Moreover, inhibition of autophagy by chloroquine increased cisplatin sensitivity in vivo. Also, the knockdown of BCAT1 or the administration of leucine activated mTOR signaling, inhibited autophagy, and increased cisplatin sensitivity in cancer cells in vivo. These findings demonstrate a new mechanism, revealing that BCAT1 decreases cisplatin sensitivity in cancer cells by inducing mTOR-mediated autophagy *via* branched-chain amino acid leucine metabolism, providing an attractive pharmacological target to improve the effectiveness of chemotherapy.

## Introduction

Cisplatin, a broad-spectrum anti-tumor chemotherapeutic agent, is used in clinical practice for the treatment of various types of tumors^1^, including hepatocellular carcinoma (HCC) and cervical cancer. However, the development of cisplatin resistance leads to treatment failure^2^. Although significant progress has been made in understanding the mechanisms of cisplatin drug resistance in human cancer, effective strategies to improve cisplatin sensitivity are still lacking. Branched-chain amino acid transaminase 1 (BCAT1) is a cytosolic enzyme that catalyzes the synthesis of α-ketoglutarates from branched-chain amino acids (BCAAs) and then to produce glutamates and branched-chain α-ketoacids^3^. It is involved in the progression of various cancer, including breast^4^, stomach^5^, esophageal^6^, lung^7^, pancreatic^8^, prostate^9^, cervical^10^ and liver cancer^11,12^. Our previous studies showed that BCAT1 expression correlated with a poor prognosis of HCC patients and promoted tumor cell growth in vitro and in vivo^11^. Moreover, BCAT1 decreased the cisplatin sensitivity of HCC cells^11^. Several studies also show that BCAT1 overexpression is associated with multi-drug resistance^3,9,13^, including bevacizumab on glioblastoma^3^, EGFR tyrosine kinase inhibitors on lung cancer^13^ and cisplatin on prostate cancer^9^. However, the exact role and mechanism of the involvement of BCAT1 in cisplatin cytotoxicity remain undefined.

BCAT1 is an enzyme responsible for initiating the catabolism of BCAAs, such as leucine (Leu), thus providing macromolecule precursors^14^. BCAAs are well-known activators of mammalian target of rapamycin (mTOR)^15^, especially Leu^16^. Moreover, the activation of mTOR inhibits autophagy flux^17^. Autophagy is an important homeostasis program that is responsible for removing dysfunctional or damaged organelles in all living cells^18^. Various stimulations, such as starvation, growth factor withdrawal, hypoxia and genotoxic or oxidative stress, can induce autophagy^19,20^. In cancer cells, autophagy is considered a double-edged sword, because it has tumor-promoting and tumor-suppressing properties^18^. Currently, the most popular view is that autophagy kills cancer cells during the tumorigenesis stage, inhibits tumor growth, and after tumorigenesis, autophagy protects cancer cell survival against various harsh environments^20^. Autophagy is suggested to mediate a cancer cell’s resistance to chemotherapy and radiation treatment^21,22^. Moreover, various studies show that autophagy contributes to cisplatin chemoresistance, and inhibiting autophagy, perhaps, is a means for overcoming cisplatin resistance^23–25^.

In this study, we demonstrated a new mechanism of cisplatin resistance mediated by BCAT1. Our results showed that the cisplatin-induced the upregulation of BCAT1, which decreased the cisplatin sensitivity of cancer cells by facilitating autophagy *via* the Leu-mediated activation of the mTOR signaling pathway.

## Results

### BCAT1 expression decreases cisplatin cytotoxicity in cancer cells

**Fig. 1:**
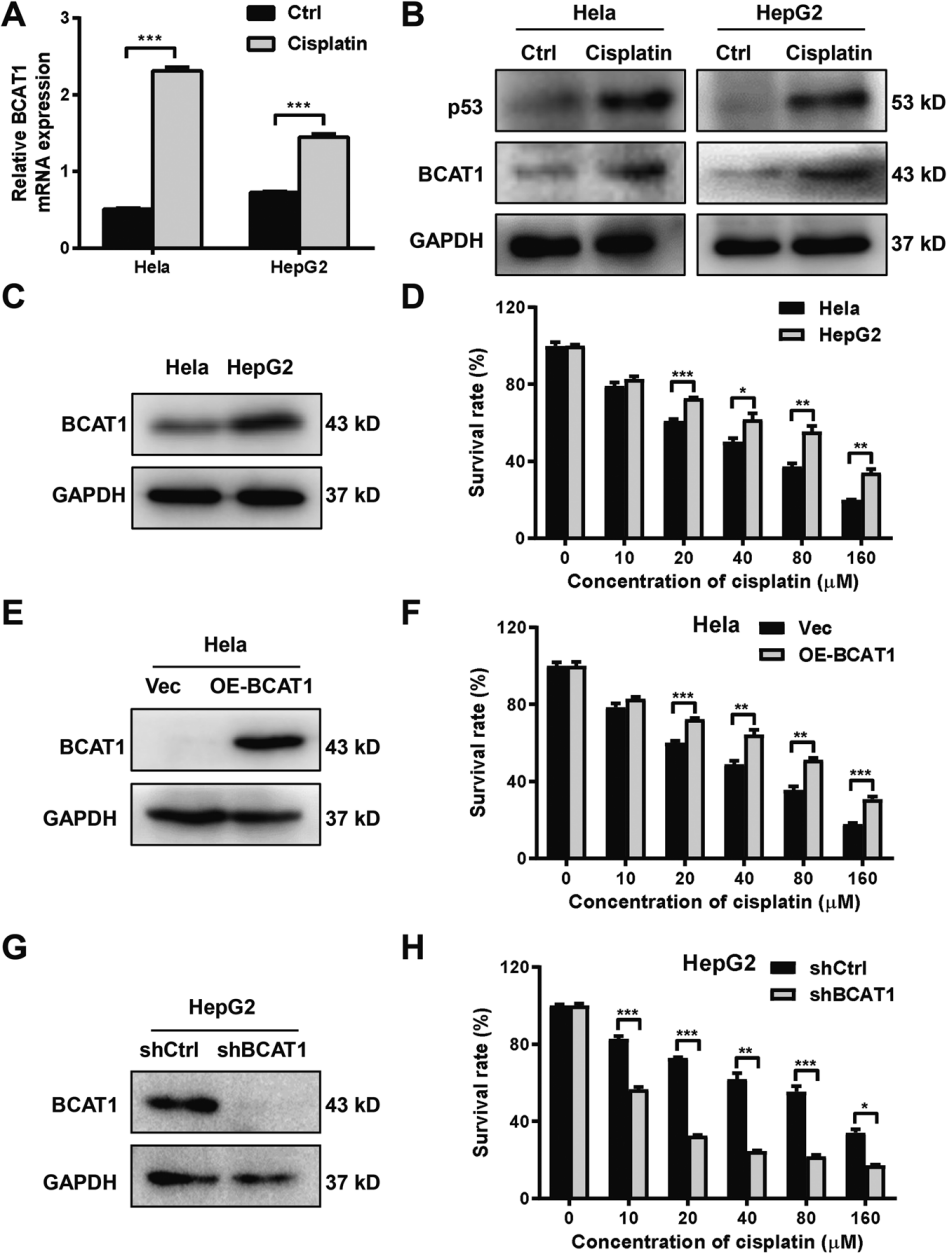
BCAT1 expression decreases cisplatin sensitivity. **A** qRT-PCR was performed to detect BCAT1 mRNA expression in Hela and HepG2 cells treated with cisplatin. Hela cells were treated with 20 μM cisplatin and HepG2 cells were treated with 10 μM cisplatin for 24 h. **B** Western blot analysis of BCAT1 and p53 protein expression in Hela and HepG2 cells treated with cisplatin (20 and 10 μM, respectively) for 24 h. **C** Western blot analysis of BCAT1 protein expression in Hela and HepG2 cell lines. **D** CCK-8 assays were performed to measure the survival rate of Hela and HepG2 cells treated with different concentrations of cisplatin for 24 h. **E** A Western blot was used to verify the increased protein level of BCAT1 in Hela cells overexpressing BCAT1. **F** CCK-8 assays were performed to examine the cytotoxicity in Hela cells overexpressing BCAT1 that were treated with different concentrations of cisplatin for 24 h. **G** A Western blot was used to verify the decreased protein level of BCAT1 in the BCAT1 knockdown HepG2 cells. **H** CCK-8 assays were used to examine the cytotoxicity in the BCAT1 knockdown HepG2 cells treated with different concentrations of cisplatin for 24 h. Vec, empty vector; Ctrl, control. Three independent experiments were performed. *P < 0.05, **P < 0.01, ***P < 0.001.

The expression levels of BCAT1 were detected by qRT-PCR and Western blotting in the cervical cancer cell line (Hela) and HCC cell lines (HepG2 and Huh-7) treated with cisplatin. The mRNA and protein expression levels of BCAT1 were significantly increased in the Hela, HepG2, and Huh-7 cells after the cisplatin treatment (Fig. 1A-B and S1A). The protein expression levels of p53 were significantly increased in Hela, HepG2, and Huh-7 cells after the cisplatin treatment (Fig. 1B and S1A). Furthermore, the BCAT1 protein level in the HepG2 cells was higher than in the Hela cells (Fig. 1C) and the HepG2 cells with a high expression of BCAT1 were less sensitive to cisplatin compared with the Hela cells with a low expression of BCAT1 (Fig. 1D). To determine the functional role of BCAT1 with respect to cisplatin sensitivity, BCAT1 was overexpressed in the Hela and Huh-7 cells, which express low levels of BCAT1 (Fig. 1C and S1B), using a lentiviral vector. In addition, BCAT1 expression was knocked down in the HepG2, which express a high level of BCAT1, using shRNA. The increased BCAT1 protein level in the Hela cells (Fig. 1E) and Huh-7 cells (Figure S1C) transfected with the lenti-BCAT1 was verified by Western blotting. The overexpression of BCAT1 significantly decreased the sensitivity of the Hela (Fig. 1F) and Huh-7 (Figure S1D) cells to cisplatin. Conversely, the knockdown of BCAT1 by shRNA greatly enhanced the cytotoxicity of cisplatin in the HepG2 cells (Fig. 1G-H).

### Cisplatin-induced autophagy decreases cisplatin cytotoxicity

**Fig. 2:**
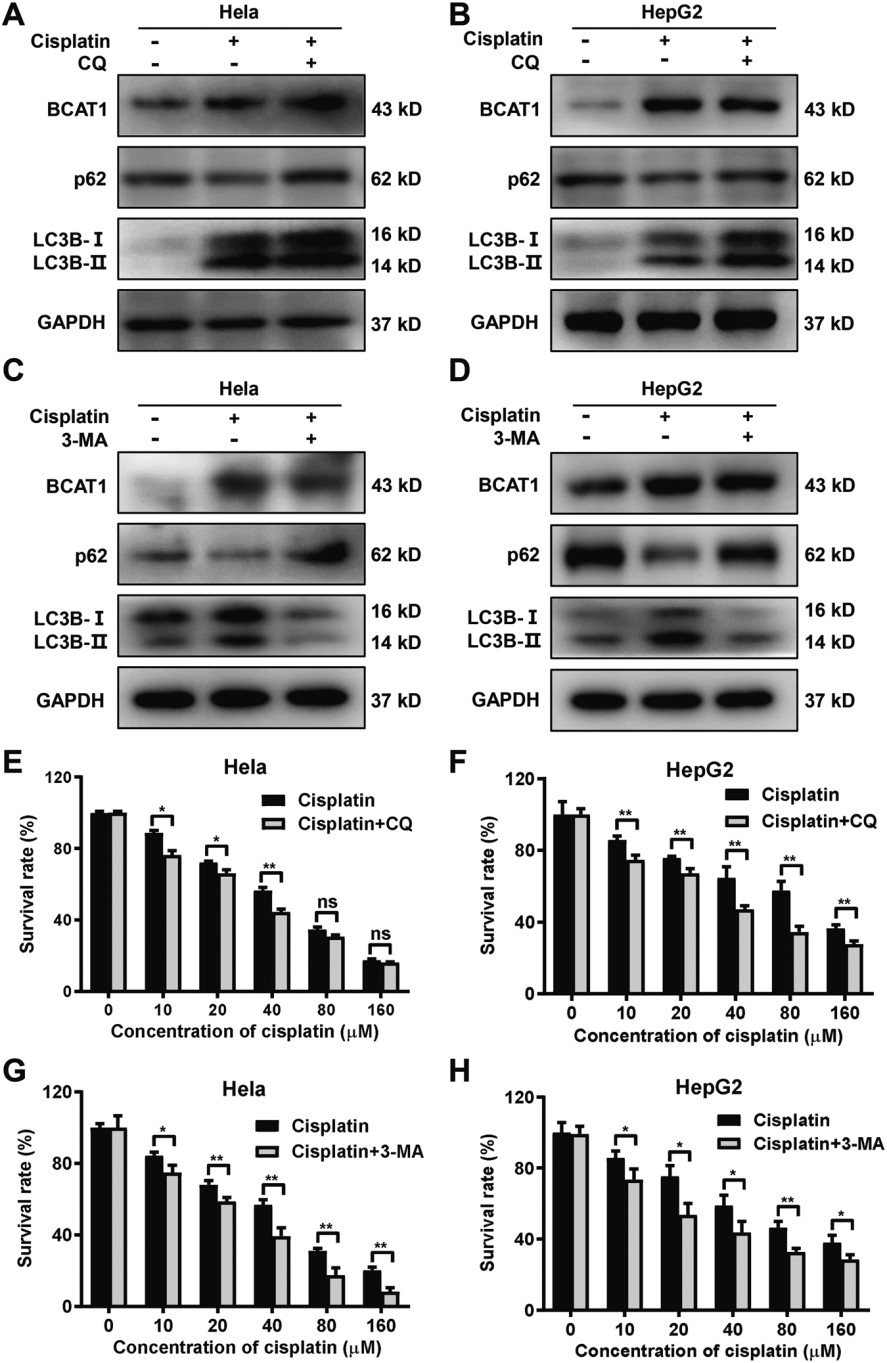
Cisplatin-induced autophagy confers decreased cisplatin cytotoxicity. **A**, **B** Western blot analysis of BCAT1, p62, and LC3-I/II protein levels in the Hela (**A**) and HepG2 (**B**) cells treated with cisplatin (20 μM and 10 μM, respectively) alone or in combination with 20 μM chloroquine (CQ) for 24 h. **C**, **D** Western blot analysis of BCAT1, p62, and LC3-I/II protein levels in the Hela (**C**) and HepG2 (**D**) cells treated with cisplatin (20 μM and 10 μM, respectively) alone or in combination with 2 mM 3-methyladenine (3-MA) for 24 h. **E**, **F** Cell viability was assessed by CCK-8 assays. Hela (**E**) and HepG2 (**F**) cells were incubated with increasing concentrations of cisplatin alone or in combination with CQ (20 μM) for 24 h. **G**, **H** Cell viability was assessed by CCK-8 assays. Hela (**G**) and HepG2 (**H**) cells were incubated with increasing concentrations of cisplatin alone or in combination with 3-MA (2 mM) for 24 h. Three independent experiments were performed. ns, not significant. *P < 0.05, **P < 0.01.

We assessed the expression level of p62 and the conversion of the LC3B-I into LC3B-II after treating the Hela, HepG2, and Huh-7 cells with cisplatin. The Western blot results showed an increase in the lipidated form of LC3 (LC3B-II) and a reduction in p62 expression after cisplatin treatment in Hela, HepG2, and Huh-7 cell lines (Fig. 2A, B and S2A-B). Although the changes in LC3B-II and p62 levels indicate the autophagic flux, the fluorescently-tagged pCMV-Cherry-GFP-LC3B vector can be used to monitor the autophagic flux more reliably^26^. Thus, the Hela or HepG2 cells were transiently transfected for 24 h with pCMV-Cherry-GFP-LC3B, and then, the cells were treated with cisplatin for 24 h. There was an increase in the mCherry positive signal after the cisplatin treatment (Figure S2C). Treatment with the autophagy inhibitor CQ or 3-MA restored the expression of p62 (Fig. 2A-D and S2A-B). Inhibition of autophagy at late stage by CQ increased the level of LC3B-II whereas suppression of autophagy initiation by 3-MA decreased the level of LC3B-II (Fig. 2A-D and S2A-B). To determine whether cisplatin-induced autophagy alters the sensitivity, Hela, HepG2, and Huh-7 cells were treated with increasing concentrations of cisplatin alone or combined with CQ or 3-MA. Interestingly, the inhibition of autophagy significantly enhanced the sensitivity of cells to cisplatin (Fig. 2E-H and S2D-E). Apoptosis and cell cycle analysis also showed that cisplatin treatment induced cell apoptosis (Figure S3A) and reduced number of cells in G2/M phase (Figure S3B). CQ or 3-MA significantly increased cisplatin-induced apoptosis (Figure S3A) and also reduced the proportion of cells in G2/M phase after cisplatin treatment (Figure S3B). These data indicated that autophagy does play a specific role in regulating cisplatin sensitivity.

### BCAT1 decreases cisplatin sensitivity by activating autophagy *via* the mTOR pathway

**Fig. 3:**
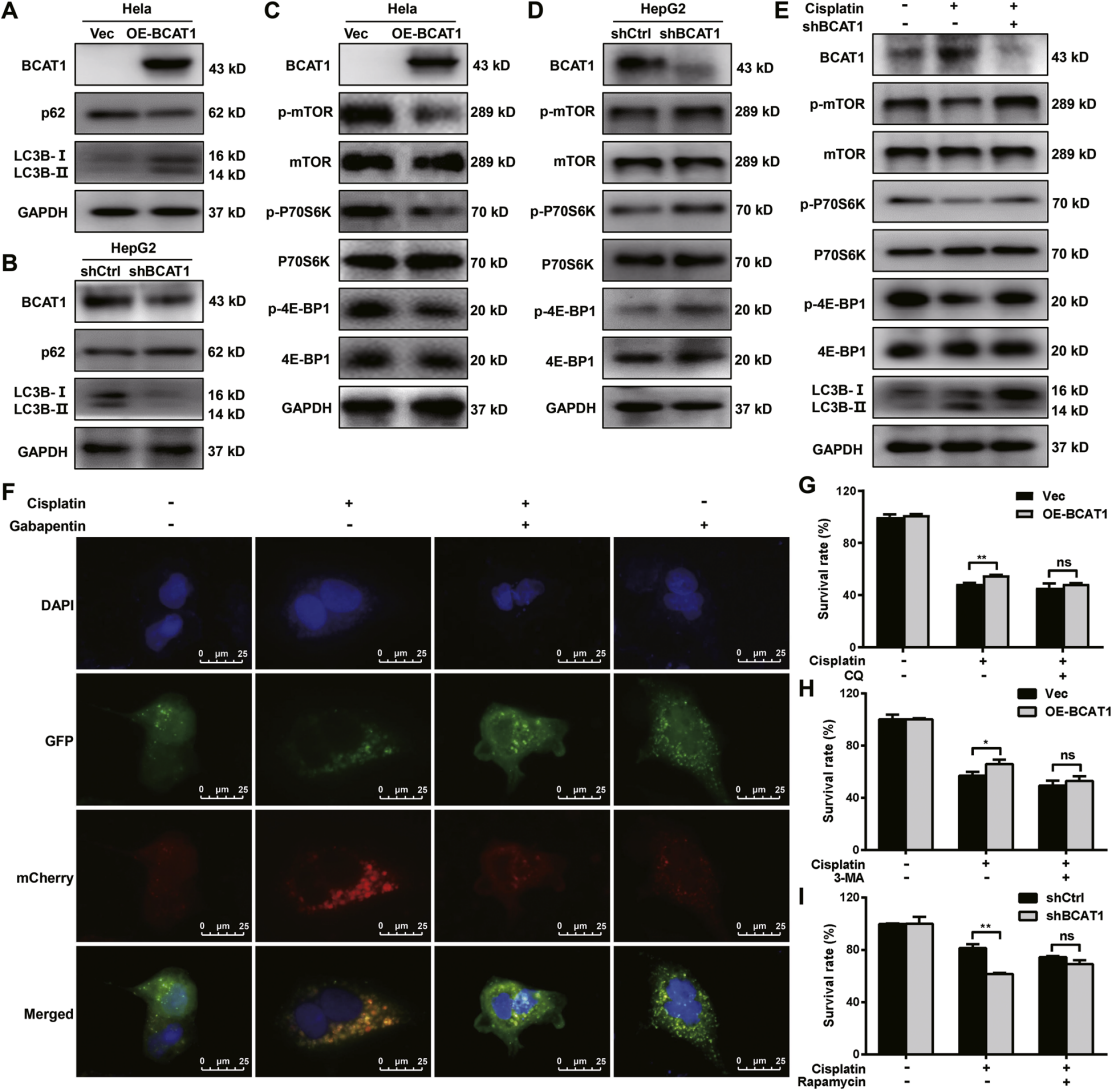
BCAT1 decreases cisplatin sensitivity by mTOR-mediated autophagy. **A** Western blot analysis of BCAT1, p62, and LC3-I/II protein levels in Hela cells overexpressing BCAT1. **B** Western blot analysis of BCAT1, p62, and LC3-I/II protein levels in BCAT1 knockdown HepG2 cells. **C** The protein levels of BCAT1, mTOR, p70S6K, and 4E-BP1 and their phosphorylated counterparts in Hela cells overexpressing BCAT1. **D** The protein levels of BCAT1, mTOR, p70S6K, 4E-BP1 and their phosphorylated counterparts in BCAT1 knockdown HepG2 cells. **E** The protein levels of BCAT1, mTOR, p70S6K, 4E-BP1 and their phosphorylated counterparts and LC3-I/II in HepG2 or BCAT1 knockdown HepG2 cells treated with cisplatin (10 μM). **F** Representative image of HepG2 cells transfected with pCMV-mCherry-GFP-LC3B. The cells were treated with cisplatin (10 μM) or gabapentin (5 mM) alone or together for 24 h. Scale bars: 25 μm. **G**, **H** Hela cells overexpressing BCAT1 were treated with cisplatin (20 μM) alone or in combination with 20 μM CQ or 2 mM 3-MA for 24 h. Cell viability was assessed by CCK-8 assays. (I) BCAT1 knockdown HepG2 cells were treated with cisplatin (10 μM) alone or plus with rapamycin (100 nM) for 24 h. Cell viability was assessed by CCK-8 assays. Vec, empty vector; Ctrl, control. Three independent experiments were performed. ns, not significant. *P < 0.05, **P < 0.01.

As shown in Fig. 3A and S4A, the overexpression of BCAT1 increases LC3B-II expression and suppresses p62 expression in Hela and Huh-7 cells, whereas the knockdown of BCAT1 decreases LC3B-II expression and increases p62 expression in HepG2 cells (Fig. 3B), indicating that BCAT1 induces autophagy in cervical cancer and HCC cells. To further explore the specific mechanism of BCAT1-regulated autophagy, we used the PEX100 Phospho Explorer Array to identify specific BCAT1 downstream effectors that regulate autophagy. The knockdown of BCAT1 expression significantly increased the phosphorylation of 4E-BP1 and p70S6K in the HepG2 cells (Figure S4B-C), indicating that the inhibition of BCAT1 expression might activate the mTOR signaling pathway. Consistent with the PEX100 Phospho Explorer Array results, the Western blot data demonstrated that the overexpression of BCAT1 decreased the phosphorylation of 4E-BP1, p70S6K, and mTOR in the Hela cells (Fig. 3C) and Huh-7 cells (Figure S4D), whereas the knockdown of BCAT1 significantly increased the phosphorylation of 4E-BP1, p70S6K and mTOR in the HepG2 cells (Fig. 3D). Moreover, the knockdown of BCAT1 restored the cisplatin-induced inhibition of the mTOR phosphorylation level and reversed cisplatin-induced autophagy (Fig. 3E). Next, the HepG2 cells were transiently transfected with pCMV-Cherry-GFP-LC3B for 24 h, and then, the cells were treated with cisplatin or the BCAT1 inhibitor gabapentin alone or together for 24 h. The results revealed that the increased mCherry positive signals induced by the cisplatin treatment were reversed by inhibiting BCAT1 with gabapentin in the HepG2 cells (Fig. 3F).

To determine the role of BCAT1-triggered autophagy in cisplatin cytotoxicity, we used autophagy inhibitors or activators to treat the Hela and Huh-7 cells with BCAT1 overexpression or the HepG2 cells with BCAT knockdown, respectively. The overexpression of BCAT1 decreased the sensitivity of Hela (Fig. 3G-H) and Huh-7 (Figure S4E-F) cells to cisplatin, and the autophagy inhibitors CQ and 3-MA reversed this effect (Fig. 3G-H and Figure S4E-F). In contrast, the mTOR inhibitor rapamycin blocked the increased cisplatin sensitivity in the HepG2 cells that was induced by the knockdown of BCAT1 (Fig. 3I). As shown in Figure S5A-C, overexpression of BCAT1 decreased cisplatin-induced apoptosis in Hela and Huh-7 cells, whereas knockdown of BCAT1 increased cisplatin-induced apoptosis in HepG2 cells. CQ treatment promoted cisplatin-induced apoptosis and reversed the effect of BCAT1 overexpression (Figure S5A-B). In contrast, rapamycin treatment suppressed cisplatin-induced apoptosis (Figure S5C). These results suggested that BCAT1 decreased the cisplatin sensitivity by regulating autophagy and by affecting the mTOR signaling pathway.

### BCAAs and leucine decrease cisplatin-mediated autophagy and increase cisplatin sensitivity

**Fig. 4:**
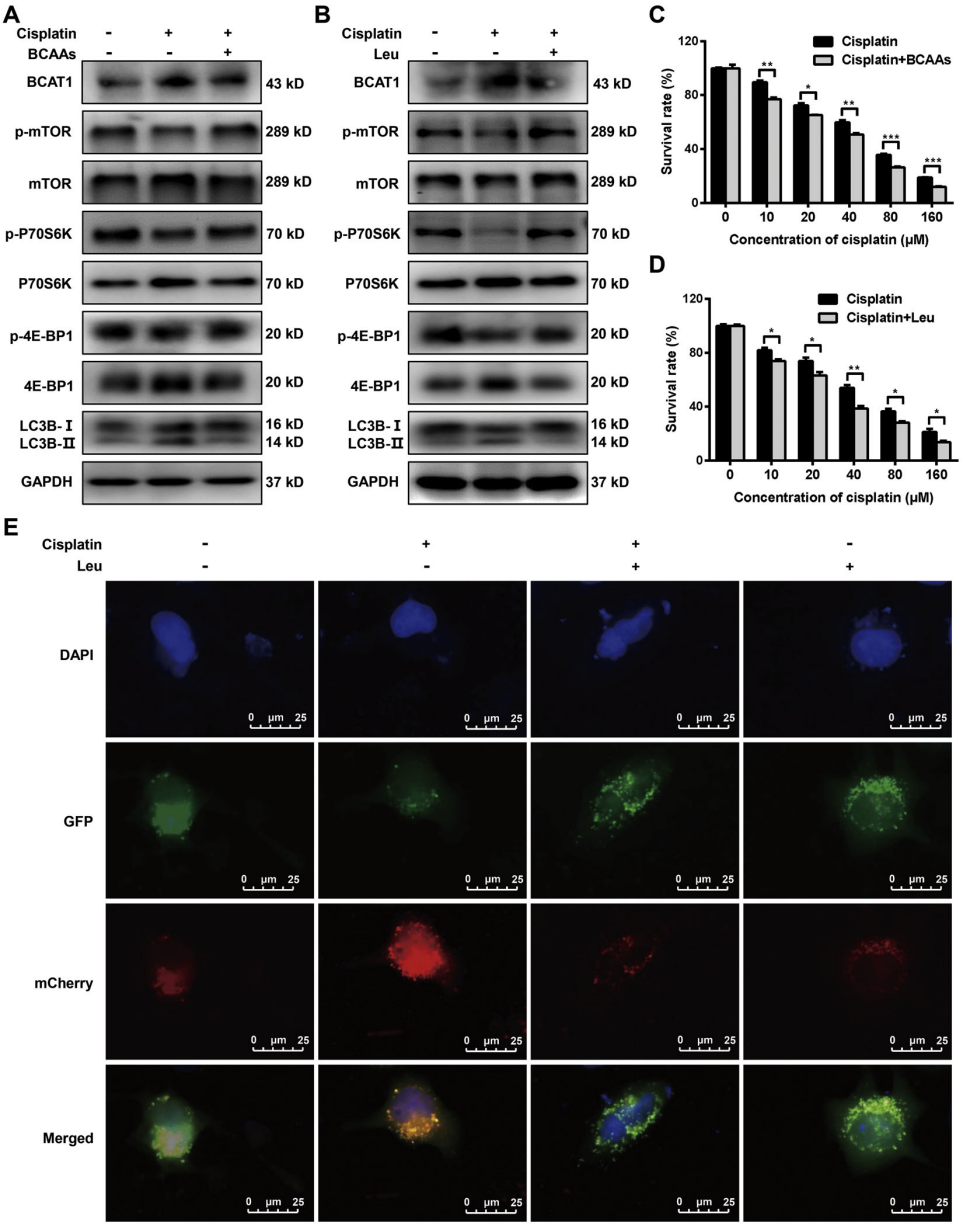
Branched-chain amino acids (BCAAs) and leucine (Leu) decrease cisplatin-mediated autophagy and increase cisplatin sensitivity. **A**, **B** Western blot analysis of the protein levels of BCAT1, mTOR, p70S6K, 4E-BP1 and their phosphorylated counterparts and LC3-I/II in Hela cells treated with cisplatin (20 μM) or in combination with BCAAs (5 mM) or Leu (2 mM) for 24 h. **C**, **D** Hela cells were treated with increasing concentrations of cisplatin alone or in combination with BCAAs (5 mM) or Leu (2 mM) for 24 h. Cell viability was assessed by CCK-8 assays. **E** Representative image of Hela cells transfected with pCMV-mCherry-GFP-LC3B. The cells were treated with cisplatin (20 μM) or Leu (2 mM) alone or together for 24 h. Scale bars: 25 μm. Three independent experiments were performed. *P < 0.05, **P < 0.01, ***P < 0.001.

To investigate the role of BCAAs or Leu in cisplatin sensitivity, we treated the Hela and Huh-7 cells with BCAAs or Leu combined with cisplatin. The BCAAs or Leu treatment reversed the decreased mTOR, 4E-BP, and p70S6K phosphorylation and the increased cisplatin-induced autophagy in the Hela (Fig. 4A, B) and Huh-7 (Figure S6A-B) cells. Moreover, the BCAAs or Leu treatment significantly increased the sensitivity of the Hela (Fig. 4C, D) and Huh-7 (Figure S6C-D) cells to cisplatin. Apoptosis and cell cycle analysis also showed that BCAAs or Leu treatment significantly increased cisplatin-induced apoptosis (Figure S3A) and reduced the proportion of cells in G2/M phase after cisplatin treatment (Figure S3B). The Hela cells were transiently transfected for 24 h with pCMV-Cherry-GFP-LC3B, and then, the cells were treated with cisplatin or Leu alone or together for 24 h. The results revealed that the increased mCherry positive signals induced by the cisplatin treatment were reversed by the addition of Leu (Fig. 4E). The above experiments indicated that BACC Leu participates in the sensitivity of the cancer cells to cisplatin.

### BCAT1 enhances autophagy and inhibits cisplatin sensitivity in cancer cells *via* leucine reduction

**Fig. 5:**
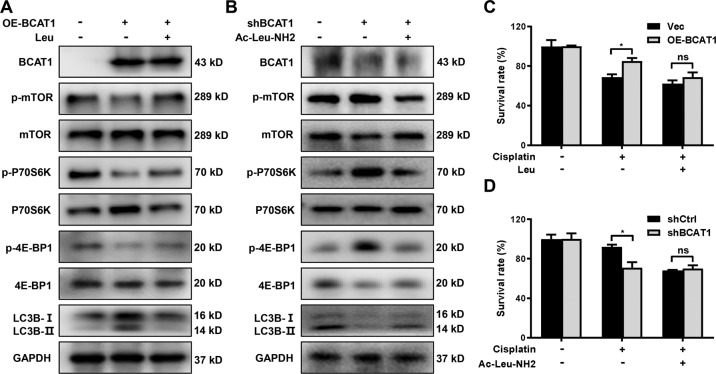
BCAT1 enhances autophagy and decreases cisplatin sensitivity *via* leucine (Leu) reduction. **A** Western blot analysis of the protein levels of BCAT1, mTOR, p70S6K, 4E-BP1 and their phosphorylated counterparts and LC3-I/II in Hela cells with BCAT1 overexpression or in combination with Leu (2 mM) treatment for 24 h. **B** Western blot analysis of the protein levels of BCAT1, mTOR, p70S6K, 4E-BP1 and their phosphorylated counterparts and LC3-I/II in HepG2 cells with BCAT1 knockdown or in combination with an Ac-Leu-NH2 (5 mM) treatment for 24 h. **C** Hela cells overexpressing BCAT1 were treated with cisplatin (20 μM) alone or in combination with Leu (2 mM) for 24 h. Cell viability was assessed by CCK-8 assays. **D** BCAT1 knockdown HepG2 cells were treated with cisplatin (10 μM) alone or in combination with Ac-Leu-NH2 (5 mM) for 24 h. Cell viability was assessed by CCK-8 assays. Vec, empty vector; Ctrl, control. Three independent experiments were performed. ns, not significant. *P < 0.05.

The Western blot results demonstrated that the Leu treatment reversed the decreased mTOR, 4E-BP and p70S6K phosphorylation levels and the increased autophagy induced by BCAT1 overexpression in the Hela (Fig. 5A) and Huh-7 (Figure S7A) cells. In comparison, the Leu antagonist, Ac-Leu-NH2, reversed the increased mTOR, 4E-BP and p70S6K phosphorylation levels and the decreased autophagy induced by BCAT1 knockdown in the HepG2 cells (Fig. 5B). Moreover, the addition of Leu blocked the decreased cisplatin cytotoxicity in the Hela (Fig. 5C) and Huh-7 (Figure S7B) cells that was induced by the overexpression of BCAT1. In contrast, the increased cisplatin sensitivity caused by BCAT1 knockdown was reversed by Ac-Leu-NH2 in the HepG2 cells (Fig. 5D). As shown in the Figure S5A-C, the treatment of Leu significantly increased the apoptotic ratio in Hela and Huh-7 cells with BCAT1 overexpression, while the effect of Ac-Leu-NH2 was just opposite in HepG2 cells with BCAT1 knockdown.

### Knockdown of BCAT1 increases cisplatin sensitivity in vivo

**Fig. 6:**
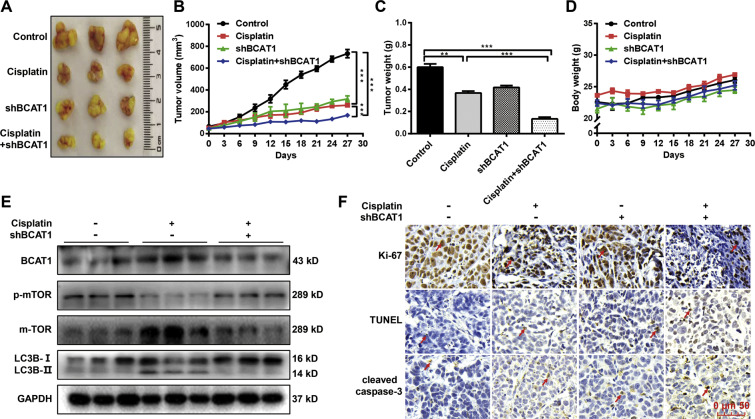
Knockdown of BCAT1 increases cisplatin sensitivity in vivo. **A** Representative images of the xenograft tumors (*n* = 5). HepG2 cells with BCAT1 knockdown by shBCAT1 or control cells were subcutaneously implanted into nude mice. Seven days later, the mice were treated daily with intraperitoneally injections of the diluents or cisplatin for 27 days. **B** Tumor volume was measured and calculated every 3 days (n = 5). **C** The final tumor weight was measured (*n* = 5). **D** The body weight of mice was measured every 3 days (*n* = 5). **E** The protein expression levels of BCAT1, LC3B-I/II, mTOR, and phosphorylated mTOR were examined in the tumor tissues by Western blot. **F** IHC staining of Ki-67 and cleaved caspase-3 and TUNEL assay. The arrow represents a positive signal. Scale bars: 50 μm. **P < 0.01, ***P < 0.001.

We further explored the role of BCAT1 in cisplatin sensitivity in vivo. As shown in Fig. 6A, B, individually, the knockdown of BCAT1 or cisplatin therapy in the HepG2 cells reduced the tumor volume. However, tumors from the cells with BCAT1 knockdown exhibited a significantly enhanced response to cisplatin, leading to a significant reduction in tumor size (Fig. 6A, B) and weight (Fig. 6C). During the treatment, no obvious change in the bodyweight of the mice was found (Fig. 6D). Moreover, the Western blot results confirmed that the knockdown of BCAT1 reversed the cisplatin-induced decrease in the mTOR phosphorylation level and increase in autophagy in tumor tissues (Fig. 6E). Immunohistochemistry analysis also showed that the knockdown of BCAT1 increased the cisplatin sensitivity, resulting in higher expression levels of cleaved caspase-3 and TUNEL, and a lower expression level of Ki-67 in tumor tissues (Fig. 6F).

### Inhibition of autophagy or the administration of Leu increases the cisplatin sensitivity of cancer cells in vivo

**Fig. 7:**
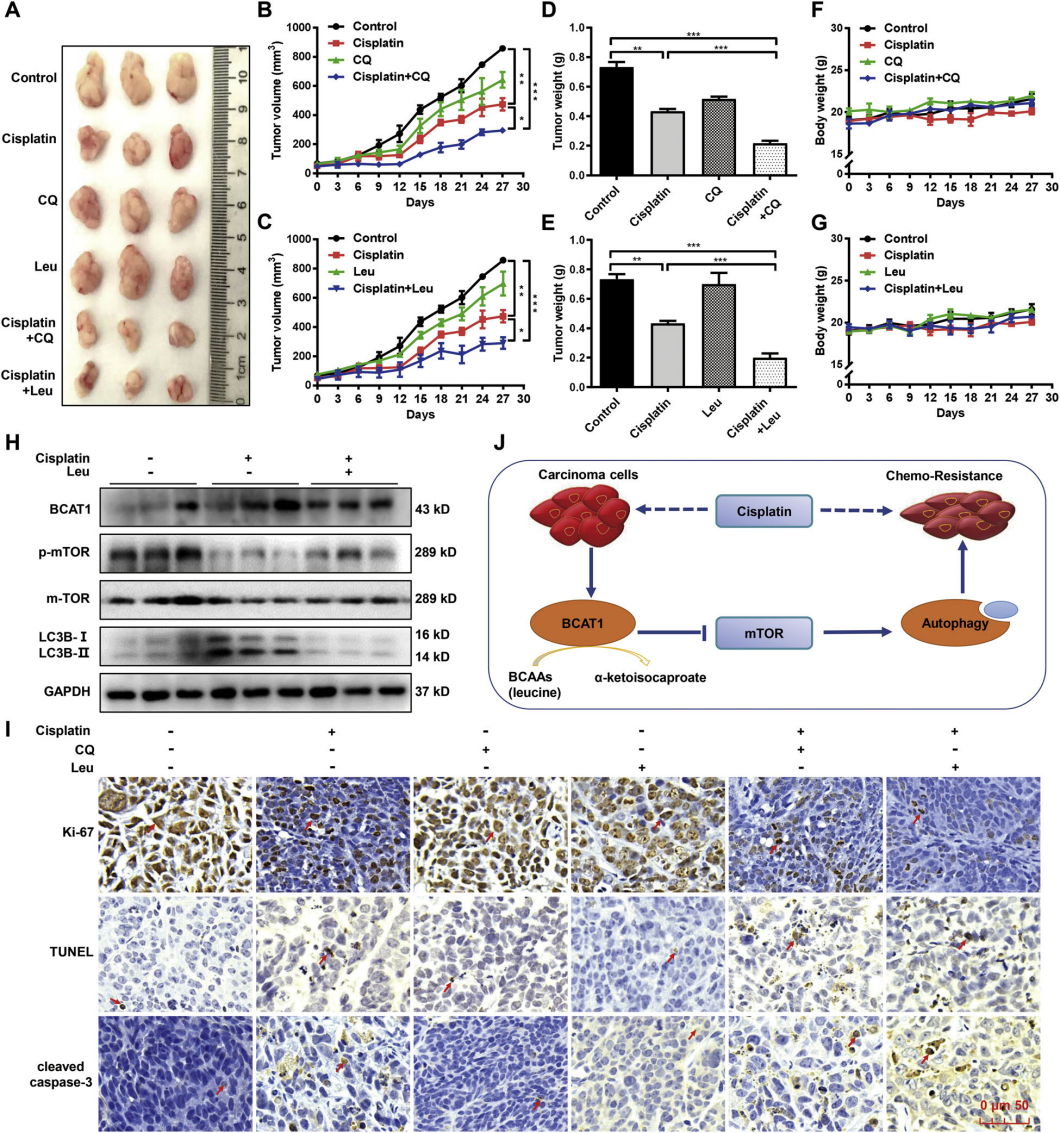
Administration of leucine (Leu) or chloroquine (CQ) increases cisplatin sensitivity in vivo. **A** Representative images of the xenograft tumors after the therapy (*n* = 5). Hela xenograft mice were treated with cisplatin, CQ, Leu, cisplatin plus CQ, or cisplatin plus Leu. **B**, **C** Tumor volume was measured and calculated every 3 days (*n* = 5). **D**, **E** The final tumor weight was measured (*n* = 5). **F**, **G** The bodyweight of mice was measured every 3 days (*n* = 5). **H** The protein expression levels of BCAT1, LC3B-I/II, mTOR, and phosphorylated mTOR were examined in the tumor tissues by Western blot. **I** IHC staining of Ki-67 and cleaved caspase-3 and TUNEL assay. The arrow represents a positive signal. Scale bars: 50 μm. (J) A schematic of the molecular mechanism. *P < 0.05, **P < 0.01, ***P < 0.001.

Next, we investigated whether Leu or autophagy was involved in the cisplatin sensitivity of cancer cells in vivo. We used the autophagy inhibitor CQ or Leu combined with the cisplatin treatment in tumor-harboring mice. The Hela cells were subcutaneously injected into the flanks of the nude mice. The tumor-harboring mice were randomly divided into 6 groups as follows: Control, Cisplatin, CQ, Leu, Cisplatin + CQ, and Cisplatin + Leu. The tumor growth curves showed that the CQ or Leu treatment significantly enhanced the response of the tumor cells to cisplatin in vivo, which resulted in a significant reduction in tumor size (Fig. 7A–C) and weight (Fig. 7D, E). However, no significant change in the bodyweight of the mice was observed (Fig. 7F, G). In addition, in accordance with the in vitro results, the Western blot data revealed that the Leu treatment reversed the cisplatin-induced decrease in the mTOR phosphorylation level and increase in autophagy (Fig. 7H). IHC analysis showed that the treatment of CQ or Leu significantly increased the cytotoxicity of the cisplatin, resulting in higher expression levels of cleaved caspase-3 and TUNEL, and a lower expression level of Ki-67 (Fig. 7I). These data suggest that the Leu treatment or inhibition of autophagy by CQ indeed altered the sensitivity of the cancer cells to cisplatin in vivo. It is worth noting that BCAT1/Leu/mTOR/Autophagy is a concise mechanism for the cisplatin sensitivity of cervical cancer and HCC cells (Fig. 7J).

## Discussion

BCAT1 expression is confirmed to be associated with cancer progression in various solid tumors^4–9,27^, including cervical cancer^10^ and HCC^12^. Increased BCAT1 expression correlates with the chemotherapeutic agent resistance^3,9,13^. For instance, Cho et al.^3^ reported that BCAT1 promoted IDH1 wild-type glioblastoma multiforme cell proliferation, invasion and increased resistance to bevacizumab treatment. In addition, Zhu et al.^9^ found that miR-218 increased chemosensitivity to cisplatin by targeting BCAT1 in prostate cancer. Moreover, Wang et al.^13^ showed that BCAT1 conferred sublethal tyrosine kinase inhibitor resistance in EGFR-mutated lung cancer cells through the H3K9 demethylation-mediated BCAA metabolism reprogramming. The above findings confirm the important role of BCAT1 in tumor drug resistance. In our previous study, we demonstrated that the exogenous expression of BCAT1 significantly promoted cell proliferation and decreased cisplatin sensitivity and increased the lipidated form of LC3 (LC3B-II) in HCC cells^11^, indicating that BCAT1 expression and autophagy have been implicated in cisplatin sensitivity. However, the molecular mechanism of how BCAT1 regulates cisplatin chemosensitivity in cancer cells remains unclear. In this study, we further revealed that BCAT1-regulated cisplatin sensitivity in cervical cancer (Hela) and HCC (HepG2 and Huh-7) cells in vitro and in vivo. Furthermore, we demonstrated a new mechanism that the cisplatin-induced up-regulation of BCAT1 decreased the cisplatin sensitivity in cervical cancer and HCC cells by regulating mTOR-mediated autophagy *via* BCAA metabolism.

Previous studies confirmed that autophagy participates in tumor drug resistance. For example, Pennati et al.^23^ found that autophagy decreased cisplatin cytotoxicity in castration-resistant prostate cancer cells. In addition, Yu et al.^24^ showed that the induction of autophagy counteracts the anticancer effect of cisplatin in acquired drug-resistant human esophageal cancer cells. Moreover, Zhao et al.^25^ demonstrated that the inhibition of autophagy sensitized gastric cancer cells to cisplatin. In this study, we revealed that cisplatin triggered autophagy in cancer cells with an increase in BCAT1 expression. We further confirmed that BCAT1 overexpression induced autophagy and decreased cisplatin sensitivity, whereas the knockdown of BCAT1 inhibited autophagy and increased cisplatin sensitivity. CQ is a classic autophagy lysosomal inhibitor. In our study, the CQ and 3-MA treatment significantly enhanced the sensitivity of the cervical cancer and HCC cells to cisplatin in vivo and in vitro and partially relieved the BCAT1-induced resistance to cisplatin. These data indicated that the cisplatin-induced up-regulation of BCAT1 reduced the sensitivity of the cervical cancer and HCC cells to cisplatin through an autophagy-related mechanism.

In order to explore the potential regulatory mechanism of BCAT1-induced autophagy, we utilized a Phospho Explorer Array to predict the potential downstream targets. The results showed that the knockdown of BCAT1 expression significantly increased the phosphorylation of 4E-BP1 and p70S6K, which are important downstream effectors of the mTOR signaling pathway^28^, suggesting that the inhibition of BCAT1 expression activates the mTOR signaling pathway. The Western blot data also demonstrated that BCAT1 overexpression suppressed the mTOR signaling pathway (by decreasing the phosphorylation of mTOR, 4E-BP1, and p70S6K). Whereas BCAT1 knockdown activated the mTOR signaling pathway (by increasing the phosphorylation of mTOR, 4E-BP1, and p70S6K). Considering that the serine/threonine kinase, mTOR pathway is the classical pathway that regulates autophagy^29^, we speculated that BCAT1 affected the cisplatin sensitivity of cancer cells by regulating autophagy through the mTOR pathway. Specifically, rapamycin, an inhibitor of the mTOR signaling pathway, decreased cisplatin sensitivity in the BCAT1 knockdown HepG2 cells.

BCAT1 is the key enzyme responsible for the transformation of BCAAs in vivo^30,31^, and BCAAs are described as a nutrient signal that activates the mTOR signaling pathway^32^. In addition, the potential beneficial effects of BCAAs have also been reported^33,34^. BCAAs administration shows a long-term inhibitory effect on carcinogenesis and improves event-free survival and overall survival^33^. Early partial replacement with BCAA-enriched nutrients in the patient’s diet may consequently improve the treatment outcome of HCC patients^34^. Therefore, we tried to explore whether BCAAs were involved in BCAT1-mediated cisplatin resistance through autophagy. Our results revealed that the BCAAs treatment reversed the cisplatin-induced mTOR signal inhibition and autophagy and restored the sensitivity of the cancer cells to cisplatin. As the most bioactive BCAA, Leu alone has the ability to activate mTOR pathway^35^. We found that Leu treatment also promoted mTOR activation, reduced autophagy flux, and increased the cisplatin cytotoxicity to cancer cells. Moreover, treatment with Leu reversed the mTOR signal inhibition and autophagy induced by BCAT1 overexpression, whereas Leu antagonist (Ac-Leu-NH2) reversed the mTOR activation and autophagy reduction caused by BCAT1 knockdown. These findings provided compelling evidence that BCAT1-induced autophagy and decreased the sensitivity of the cancer cells to cisplatin *via* a Leu-regulated mTOR signaling pathway.

However, there are several limitations of our study. (1) Further studies with the cisplatin-resistant cell lines and patient samples are needed to corroborate our conclusion; (2) In our study, tumor cell lines were directly transplanted into immunocompromised mice to form Cell line Derived Xenograft (CDX) models. However, CDX models have been shown to be limited predictors of clinical outcome^36^. The complexity of tumor microenvironment, host immune responses, and tumor heterogeneity pose challenges to clinical success. Alternatively, tumors harvested from Genetically Engineered Mice (GEM) can be transplanted and expanded into fully immunocompetent syngeneic hosts, forming GEM-Derived Allograft (GDA) models^36^. The models can be used for the preclinical studies of chemotherapeutic and small-molecule drugs, but also for the preclinical studies of various immunotherapy drugs. (3) In our study, we found that cisplatin treatment can up-regulate the expression of BCAT1, and BCAT1 plays an important role in cisplatin sensitivity. However, whether this phenomenon exists in other DNA damaging agents or chemotherapeutic drugs remains to be investigated.

In conclusion, we discovered the key role of cisplatin-induced BCAT1 in the regulation of autophagy. Our results confirmed that BCAT1 enhances the mTOR-mediated autophagy *via* BACC Leu metabolism in cancer cells, which finally leads to a reduced cisplatin sensitivity. These findings strongly suggest that BCAT1/Leu/mTOR/autophagy signaling represents a novel pathway regulating cisplatin sensitivity, thus providing an attractive pharmacological target for chemotherapy to treat cancer.

## Materials and methods

### Cell lines

Human cervical cancer cell line (Hela), HCC cell lines (Huh-7 and HepG2), and human Embryonic Kidney 293 T cell line (HEK293T) were obtained from the Chinese Academy of Sciences (Shanghai, China). All cells were cultured in DMEM (Gibco, Grand Island, NY, USA) supplemented with 10% fetal bovine serum (Sigma-Aldrich, St. Louis, MO, USA) and 1% antibiotic-antimycotic (Sigma-Aldrich). The cells were incubated in a humidified atmosphere at 37 °C with 5% CO_2_.

### Reagents

The following small molecules were used: cisplatin and Leu (Sigma-Aldrich), chloroquine (CQ), rapamycin, gabapentin, and 3-methyladenine (3-MA) (MedChemExpress, Princeton, NJ, USA), Ac-Leu-NH2 (BACHEM, Switzerland), BCAAs (Anhui BBCA Pharmaceuticals Co., Ltd., China). The cisplatin, CQ, gabapentin, 3-MA, Leu, Ac-Leu-NH2, and BCAAs were dissolved in phosphate buffer saline, and rapamycin was dissolved in dimethyl sulfoxide (Sigma-Aldrich).

### Lentiviral vectors and transduction

Lentiviral vectors expressing the BCAT1 gene or a short-hairpin RNA (shRNA) targeting BCAT1 (shBCAT1) were constructed as previously described^11^. The lentiviral particles were produced as described previously^11^. The cells were seeded into 24-well plates at a density of 2 × 10^4^/well and were transduced with the lentivirus.

### Western blot analysis

The cells or tissue was lysed in RIPA buffer (Thermo Fisher Scientific, Waltham, MA, USA) and protein concentration was measured using the Pierce BCA Protein Assay Kit (Thermo Fisher Scientific). The total protein (40 μg) was electrophoresed using 12% SDS-PAGE and was then transferred onto polyvinylidene difluoride membranes (Millipore, Bedford, MA, USA). The membranes were blocked in 5% skim milk (BioFroxx, Germany) and were incubated at 4°C with primary antibodies overnight. Subsequently, the membranes were incubated with horseradish peroxidase-conjugated secondary antibody at room temperature for 1 h. The samples were visualized using an enhanced chemiluminescence detection and were analyzed using IMAGE LAB software (Bio-Rad). The following primary antibodies were used: anti-GAPDH (Cat. # 5174), anti-LC3B (Cat. # 3868), anti-SQSTM1/p62 (Cat. # 39749), anti-mTOR (Cat. # 2983 S), anti-phosphorylated mTOR (Ser2448) (Cat. # 5536 S), anti-4E-BP1 (Cat. # 9644 T), anti-phosphorylated 4E-BP1 (Thr70) (Cat. # 9455), anti-p70 S6 Kinase (Cat. # 2708 T) and anti-phosphorylated p70 S6 Kinase (Thr421/Ser424) (Cat. # 9204) (all were purchased from Cell Signaling Technology, Danvers, MA, USA), anti-p53 was purchased from Abcam (Cat. # ab 131442, Cambridge, MA, USA), and anti-BCAT1 was purchased from BD Biosciences (Cat. # 611271, San Jose, CA, USA). The HRP-conjugated secondary antibodies were from Thermo Fisher Scientific (Cat. # 31460, Cat. # 31430, Waltham, MA, USA). Three independent experiments were performed.

### Real-time quantitative PCR (qPCR)

The total RNA was extracted from the cells using TRIzol (Thermo Fisher Scientific), and the complementary DNA was synthesized using the PrimeScript reverse transcription reagent Kit (Takara, Japan) according to the manufacturer’s protocol. The real-time quantitative PCR (qPCR) was performed using TB Green Premix Ex Taq (Takara) on the ABI QuantStudio 5 real-time PCR system (Applied Biosystems, Warrington, U.K.). The relative quantitation was expressed by using the Ct values, which were determined from triplicate reactions for each target gene and glyceraldehyde-3-phosphate dehydrogenase (GAPDH). The triplicate Ct values were averaged, and the GAPDH Ct was subtracted to obtain the ΔCt. The 2^-ΔΔCt^ method was utilized to calculate the gene expression values. The following forward and reverse primers were used, respectively: BCAT1: 5′- CAA CTA TGG AGA ATG GTC CTA -3′ and 5′- TGT CCA GTC GCT CTC TTC TCT -3′; and GAPDH: 5′- CCA GCC GAG CCA CAT CGC TC -3′ and 5′- ATG AGC CCC AGC CTT CTC CAT -3′. Three independent experiments were performed.

### Cell viability assay

The cell counting kit-8 (CCK-8) assay was used to assess the cell viability. According to the manufacturer’s instructions, the cells (2500 per well) were seeded into 96-well plates in a volume of 100 μL of complete medium. After 24 h, the treatment regimen of the various drug combinations was added. Then, after 24 h of incubation, 10 μL of the CCK8 (Dojindo, Japan) solution was added to each well, and the plate was incubated for 3 h. The absorbance value was measured at a wavelength of 450 nm. For the BCAAs, Leu, or Ac-Leu-NH2 treatment experiments, the cells were serum-starved 12 h prior to the treatment. Three independent experiments were performed.

### Cell apoptosis assay

Cell apoptosis was determined using Annexin V-FITC/PI apoptosis kit (Multi Sciences) or Annexin V-APC/7-AAD apoptosis kit (BD Pharmingen, San Diego, CA, USA). According to the manufacturer’s instructions, cells (5 × 10^5^) and incubated with Annexin V and PI or 7-AAD in the dark for 15 min. Apoptotic cells were detected with a flow cytometer (Beckman Coulter Inc, Miami, FL, USA). Three independent experiments were performed.

### Cell cycle assay

The cell cycle was checked using a 7-AAD nucleic acid dye (BD Pharmingen). Briefly, cells (1 × 10^6^ per well) were seeded in the serum-free media for 24 h and then treated with reagents for 24 h. After treatment, cells were harvested at a density of 5 × 10^5^ cells/mL and fixed overnight at 4 °C in 70% ethanol. Later, cells were washed with PBS and incubated with 7-AAD in the dark for 20 min. The DNA content of stained cells were analyzed with a flow cytometer (Beckman Coulter Inc). Three independent experiments were performed.

### Detection of autophagic flux

The plasmid pCMV-mCherry-GFP-LC3B was purchased from Beyotime Biotechnology (Shanghai, China). The cells were transiently transfected with the pCMV-mCherry-GFP-LC3B vector for 24 h using the Lipofectamine 3000 Reagent (Invitrogen) and were then treated with the different drugs for 24 h. Autophagic flux was determined by fluorescence microscopy (Leica, Wetzlar, Germany). For the Leu treatment experiments, the cells were serum starved 12 h prior to the treatment. Three independent experiments were performed.

### Phospho explorer antibody microarray analysis of the phosphoproteins

A PEX100 Phospho Explorer Array (Full Moon BioSystems Inc., Sunnyvale, CA, USA) was used to explore the BCAT1 downstream effectors. The extent of the protein phosphorylation was measured as a ratio of the “phospho” and “non-phospho” values. For the chip image obtained by scanning, the original data were analyzed with GenePix Pro 6.0 software (Axon Instruments, Foster City, CA, USA).

### Immunohistochemistry (IHC) and TUNEL assay

Paraffin-embedded tumor tissue sections were taken for IHC analysis as described previously^11^. The following primary antibodies were used: anti-Ki-67 was purchased from Abcam, and anti-cleaved caspase-3 was purchased from Saville Biotechnology (Wuhan, China). The secondary antibody was from Saville Biotechnology. Cell apoptosis in tumor tissue sections (4 μm) were evaluated with a DAB (SA-HRP) TUNEL Cell Apoptosis Detection Kit (Saville Biotechnology).

### Animal model experiments

All the animal experiments were approved by the Animal Experimental Ethics Committee of Wenzhou Medical University. Male BALB/c nude mice (3–4 weeks old, 16–20 g) were purchased from the Beijing Vital River Laboratory Animal Technology Co., Ltd. All the mice were given one week to adapt to the new environment before further experimentation. A tumor xenograft model was conducted by subcutaneously implanting isogenic HepG2 cells (5 × 10^6^/0.2 ml) transduced with lenti-shControl or lenti-shBCAT1 into athymic nude mice. The mice were randomly divided into four groups (5 mice/group) as follows: Control, Cisplatin, shBCAT1, and Cisplatin + shBCAT1 groups. We also established a tumor xenograft model by subcutaneously implanting 5 × 10^6^/0.2 ml Hela cells into athymic nude mice. After 7 days, the mice were randomized into the control or treatment groups (5 mice/group) as follows: Control, Cisplatin, CQ, Leu, Cisplatin + CQ, and Cisplatin + Leu groups. The mice were treated daily with intraperitoneally injections of the diluents or cisplatin (2 mg·kg^−1^) and/or CQ (20 mg kg^−1^) or Leu (150 mg kg^−1^) for 27 days. During the drug treatments, the subcutaneous tumor size of the nude mice was calculated using the formula (0.5 × length × width^2^) and the bodyweight of mice was checked every three days. After 27 days of treatment, the nude mice were sacrificed, and the tumors were removed, weighed. Three representative xenograft tumors in each group were taken pictures.

### Statistical analysis

All the data are proven by three independent experiments and expressed as the means ± standard deviation (SD). The statistical analyses were performed using GraphPad Prism 7.0 software (San Diego, CA, USA). No samples or mice were excluded from the analysis as outliers. The variance was similar between the groups that are being statistically compared. Differences between groups were analyzed by using student’s t-test or one-way ANOVA. No samples or mice were excluded from the analysis as outliers. *P* < 0.05 was considered statistically significant. The quantitative analyses of Western blot and TUNEL staining results are provided in Supplementary data (Figure S8-9).

## Supplementary information

Supplemental material
