# Supplementary material for: BCAT1 decreases the sensitivity of cancer cells to cisplatin by regulating mTOR-mediated autophagy via branched-chain amino acid metabolism
Source: Cell Death Dis. 2021 Feb 10;12(2):169. doi: 10.1038/s41419-021-03456-7 (PMC7876012; doi:10.1038/s41419-021-03456-7)
Supplement: Supplementary file 1 — Supplemental material [file 41419_2021_3456_MOESM1_ESM.pdf]

Supplemental data

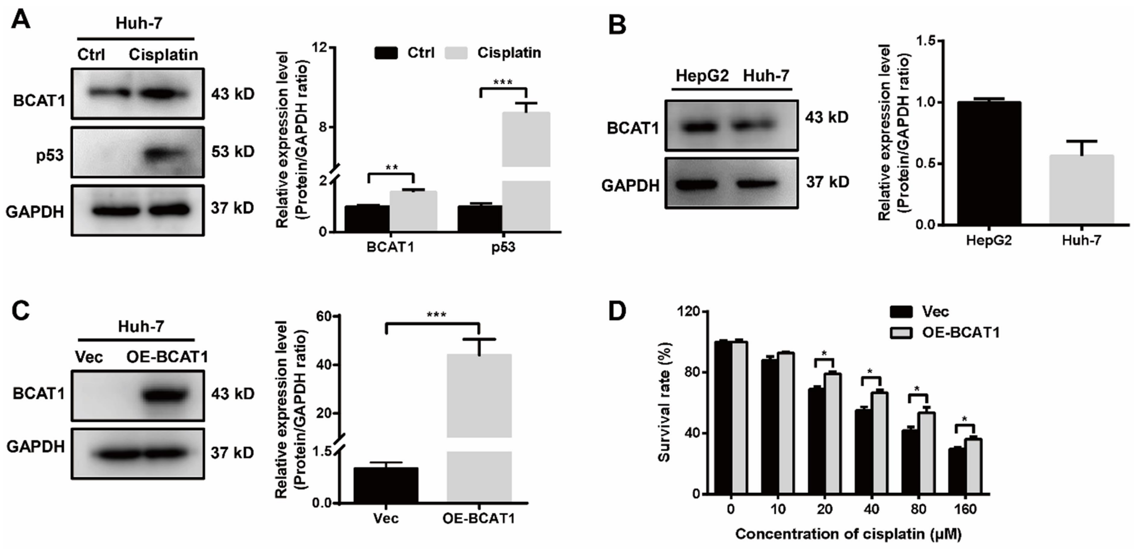

Figure S1. BCAT1 expression decreases cisplatin sensitivity in Huh-7 cells. (A) The BCAT1 expression levels were detected by Western blotting in Huh-7 cells treated with cisplatin (20 μM) or control for 24 h, and GAPDH was used as the internal control. (B) Western blot analysis of BCAT1 protein expression in HepG2 and Huh-7 cell lines, and GAPDH was used as control. (C) A Western blot was used to verify the increased protein level of BCAT1 in Huh-7 cells overexpressing BCAT1, and GAPDH was used as the internal control. (D) CCK-8 assays were performed to examine the cytotoxicity in Huh-7 cells overexpressing BCAT1 that were treated with different concentrations of cisplatin for 24 h. Three independent experiments were performed. $*P < 0.05$, $**P < 0.01$, $***P < 0.001$.

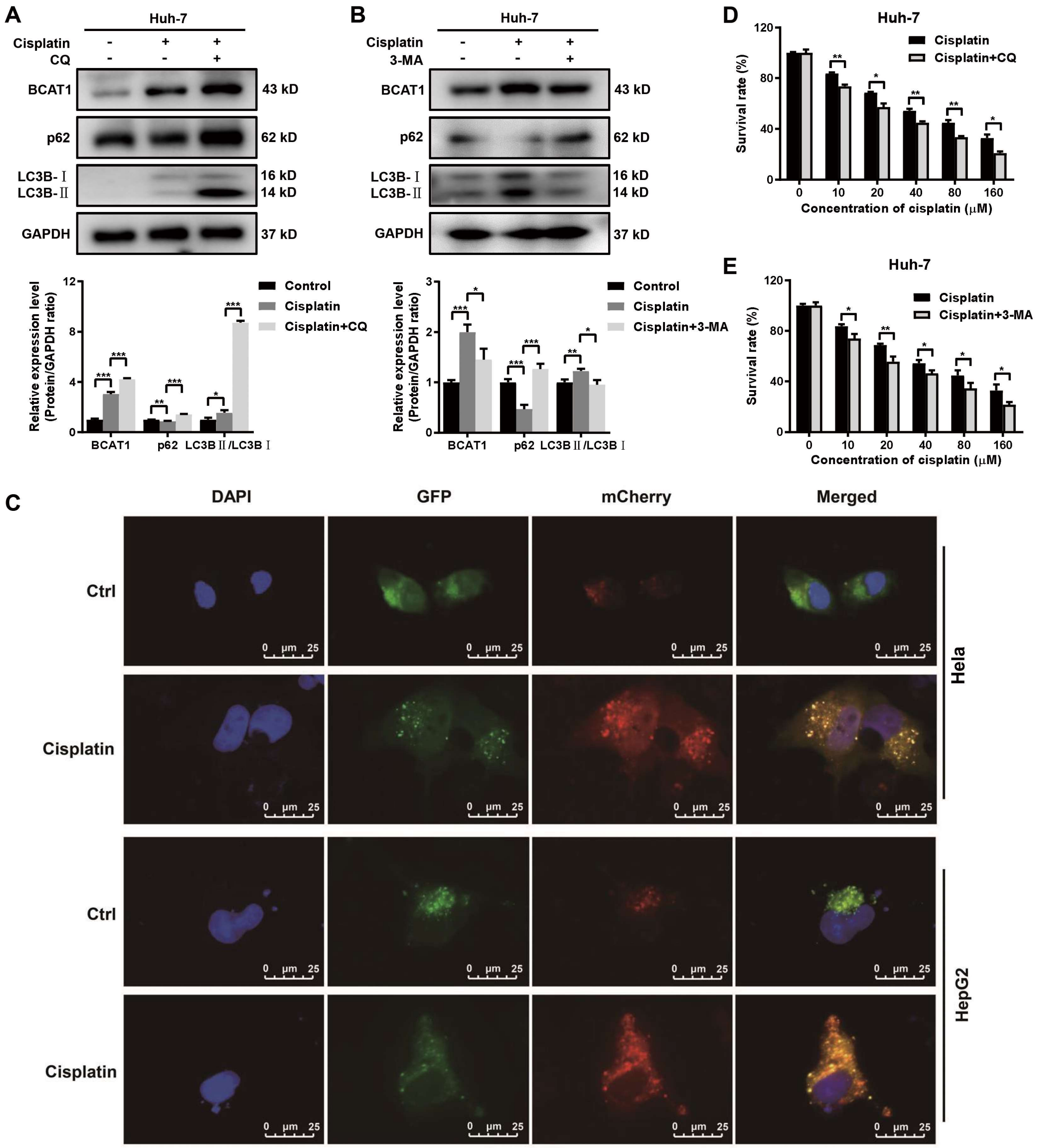

Figure S2. Cisplatin-induced autophagy confers decreased cisplatin cytotoxicity in Huh-7 cells. (A-B) Western blot analysis of BCAT1, p62 and LC3-I/II protein levels in Huh-7 cells treated with cisplatin (20 μM) alone or in combination with 20 μM chloroquine (CQ) or 2 mM 3-methyladenine (3-MA) for 24 h, GAPDH was used as the internal control. (C) Representative image of Hela and HepG2 cells transfected with pCMV-mCherry-GFP-LC3B. The cells were treated with cisplatin (20 μM and 10 μM, respectively) for 24 h. (D-E) Cell

viability was assessed by CCK-8 assays. Huh-7 cells were incubated with increasing concentrations of cisplatin alone or in combination with CQ (20 μM) or 3-MA (2 mM) for 24 h. Three independent experiments were performed. *$P < 0.05$, **$P < 0.01$, ***$P < 0.001$.

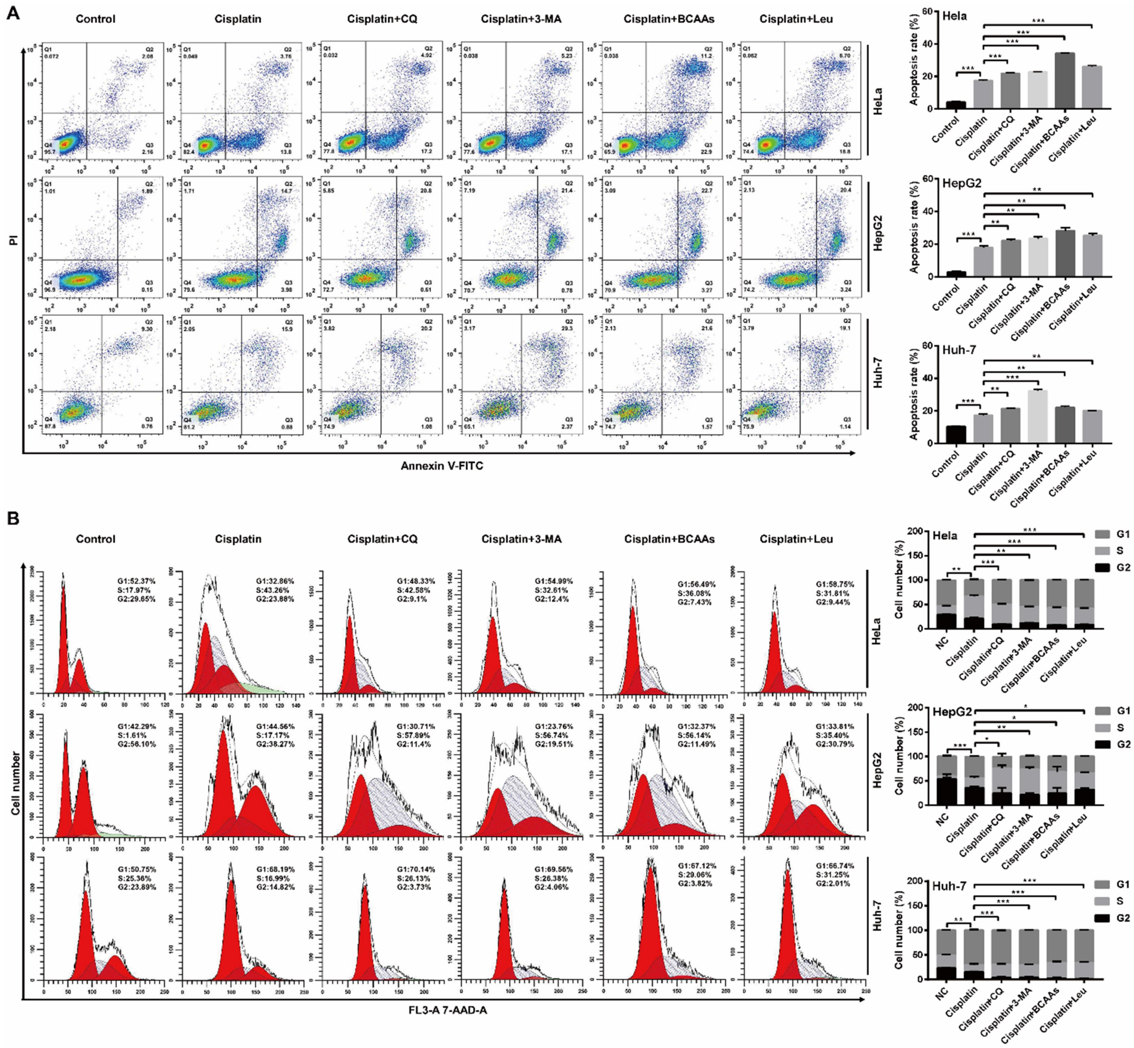

Figure S3. Apoptosis assay and cycle assay in cancer cells. (A) The apoptotic ratio in Hela, HepG2 and Huh-7 cells treated with cisplatin (20 μM, 10 μM and 20 μM, respectively) alone or plus with 20 μM CQ, 2 mM 3-MA, 5mM BCAAs, or 2 mM Leu for 24 h. (B) The cycle in Hela, HepG2 and Huh-7 cells treated with cisplatin (20 μM, 10 μM and 20 μM, respectively) alone or in combination with 20 μM CQ, 2 mM 3-MA, 5mM BCAAs, or 2 mM Leu for 24 h. Three independent experiments were performed. *$P < 0.05$, **$P < 0.01$, ***$P < 0.001$.

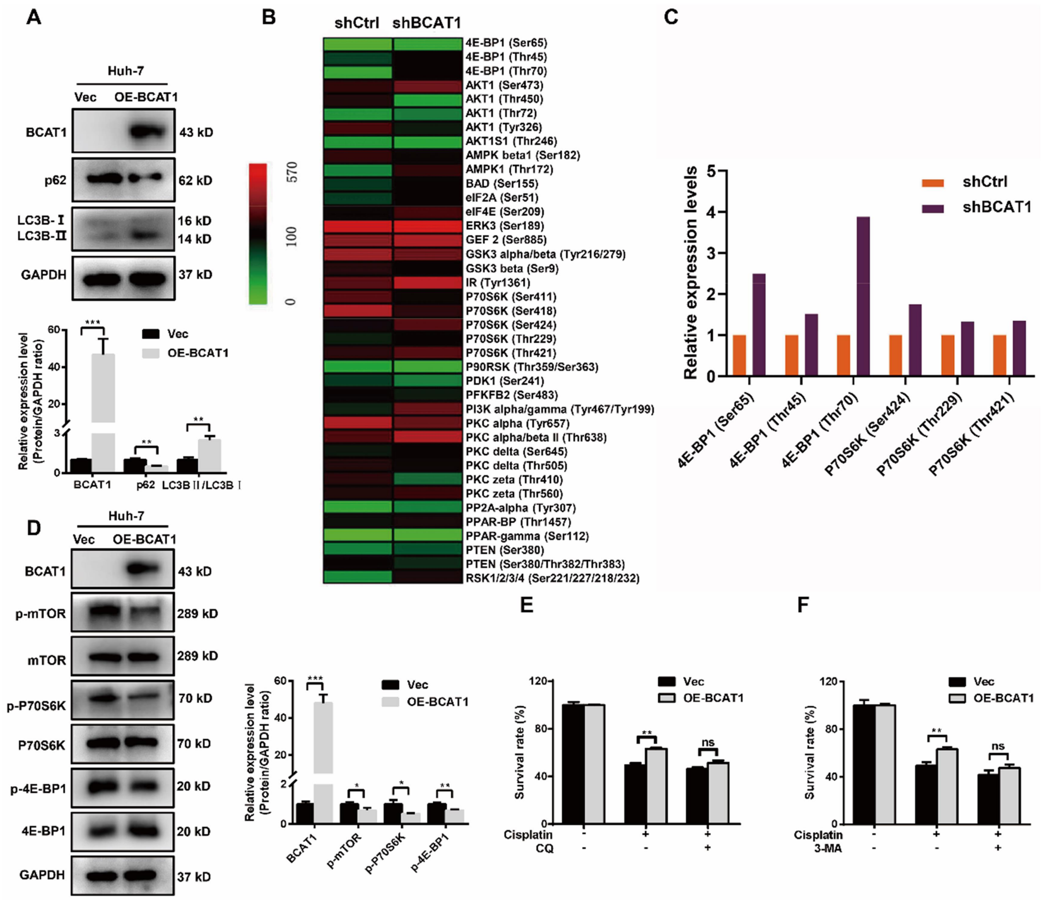

Figure S4. BCAT1 decreases cisplatin sensitivity by mTOR-mediated autophagy. (A) Western blot analysis of BCAT1, p62 and LC3-I/II protein levels in Huh-7 cells overexpressing BCAT1, GAPDH was used as the internal control. (B) A heatmap of the PEX100 Phospho Explorer Array of HepG2 cells knocking down BCAT1. (C) Quantitative analysis of the heatmap on the phosphorylation levels of 4E-BP1 and p70S6K. (D) The protein levels of BCAT1, mTOR, p70S6K and 4E-BP1 and their phosphorylated counterparts in Huh-7 cells overexpressing BCAT1, GAPDH was used as the internal control. (E-F) Huh-7 cells overexpressing BCAT1 were treated with cisplatin (20 μM) alone or plus with 20 μM CQ or 2 mM 3-MA for 24 h. Cell viability was assessed by CCK-8 assays. Three independent experiments were performed. ns, not significant. *$P < 0.05$, **$P < 0.01$, ***$P < 0.001$.

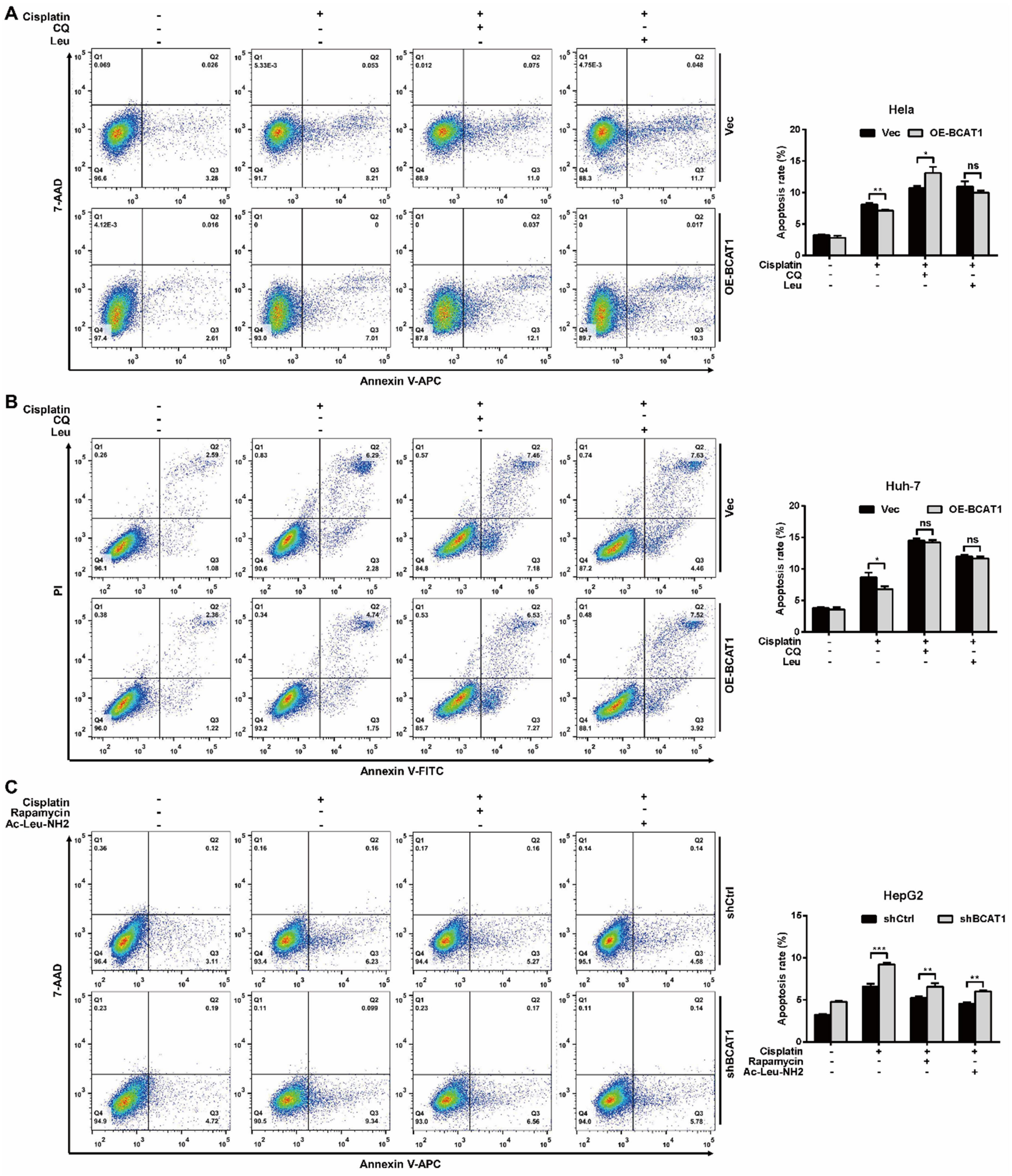

Figure S5. Apoptosis assay in cancer cells. (A-B) Hela and Huh-7 cells overexpressing BCAT1 were treated with cisplatin (20 μM) alone or in combination with 20 μM CQ or 2 mM leucine (Leu) for 24 h. Apoptotic cell ratio was assessed. (C) HepG2 cells with BCAT1 knockdown were treated with cisplatin (10 μM) alone or plus with rapamycin (100 nM) or Ac-Leu-NH2 (5 mM) for 24 h. Apoptotic ratio was assessed. Three independent experiments were performed. ns, not significant. $^{*}P < 0.05$, $^{**}P < 0.01$, $^{***}P < 0.001$.

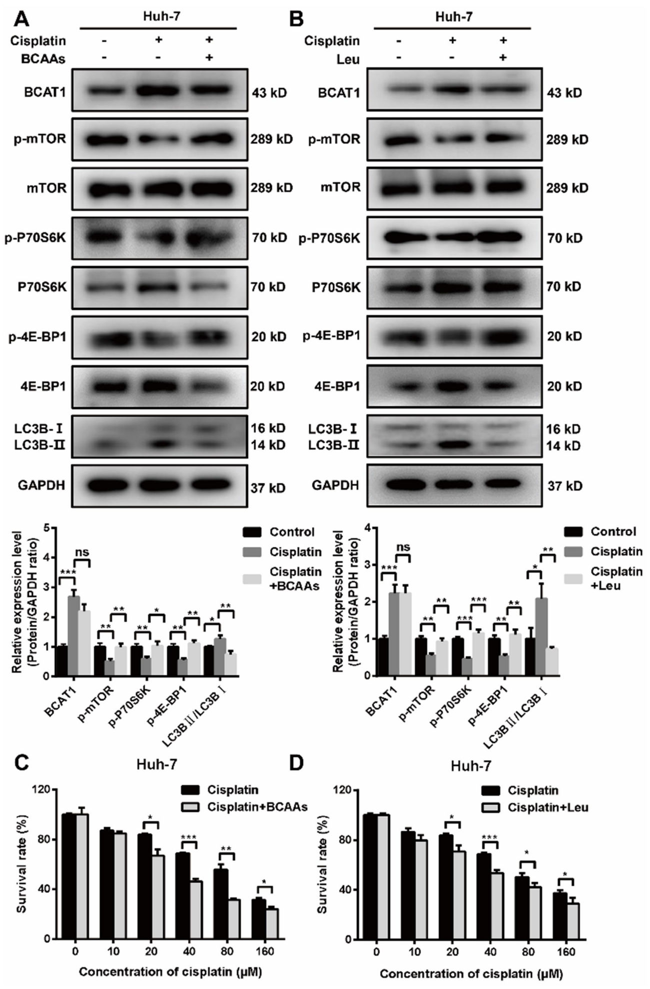

Figure S6. Branched-chain amino acids (BCAAs) and leucine (Leu) decrease cisplatin-mediated autophagy and increase cisplatin sensitivity in Huh-7 cells. (A-B) Western blot analysis of the protein levels of BCAT1, mTOR, p70S6K, 4E-BP1 and their phosphorylated counterparts and LC3-I/II in Huh-7 cells treated with cisplatin (20 μM) alone or in combination with BCAAs (5 mM) or Leu (2 mM) for 24 h, GAPDH was used as the internal control. (C-D) Huh-7 cells were treated with increasing concentrations of cisplatin alone or in combination with BCAAs (5 mM) or Leu (2 mM) for 24 h. Cell viability was assessed by CCK-8 assays. Three independent experiments were performed. ns, not significant. *$P < 0.05$, **$P < 0.01$, ***$P < 0.001$.

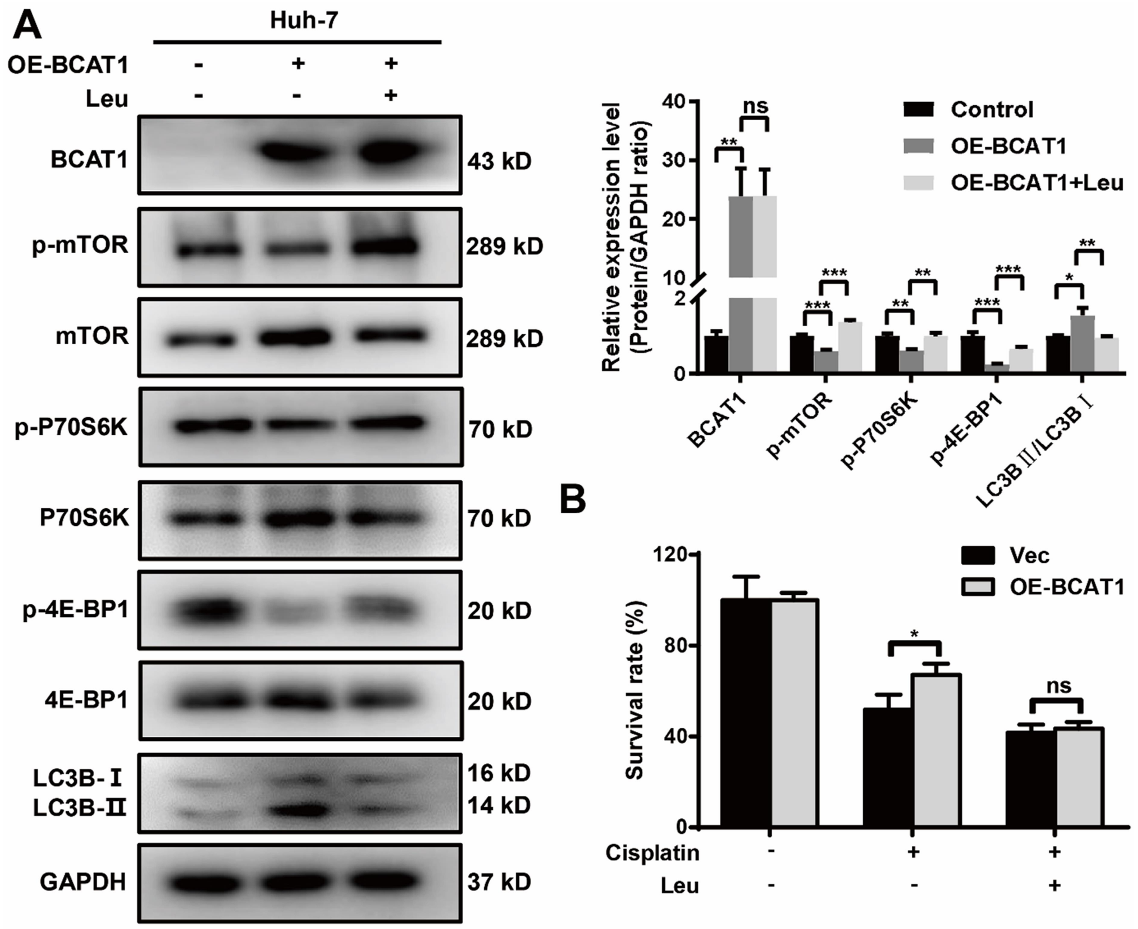

Figure S7. BCAT1 enhances autophagy and inhibits cisplatin sensitivity in Huh-7 cells via leucine reduction. (A) The protein levels of BCAT1, mTOR, p70S6K and 4E-BP1 and their phosphorylated counterparts and LC3-I/II in Huh-7 cells with BCAT1 overexpression or plus with Leu (2 mM) treatment for 24 h, GAPDH was used as the internal control. (B) Huh-7 cells overexpressing BCAT1 were treated with cisplatin (20 μM) alone or plus with 2 mM Leu for 24 h. Cell viability was assessed by CCK-8 assays. Three independent experiments were performed. ns, not significant. **P* < 0.05, ***P* < 0.01, ****P* < 0.001.

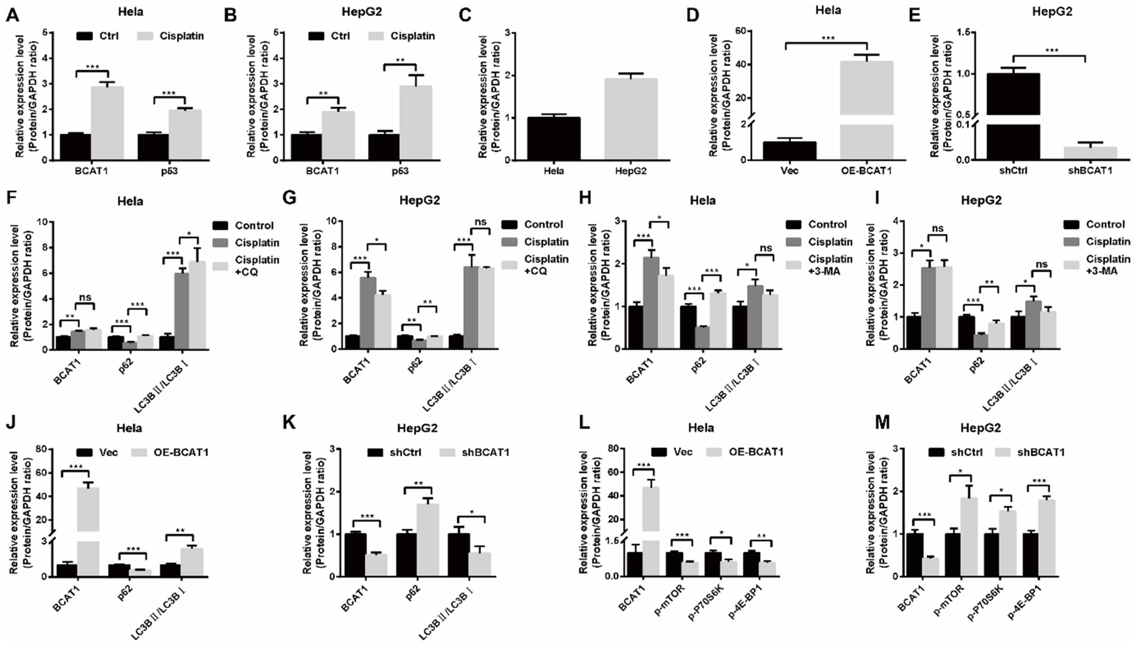

Figure S8. Qualification and statistics for the results of Western blot. (A-B) Quantitative analysis for Figure 1B. The BCAT1 and p53 protein expression in Hela and HepG2 cells treated with cisplatin (20 and 10 μM, respectively) for 24 h. (C) Quantitative analysis for Figure 1C. BCAT1 protein expression in Hela and HepG2 cell lines. (D) Quantitative analysis for Figure 1E. The increased protein level of BCAT1 in Hela cells overexpressing BCAT1. (E) Quantitative analysis for Figure 1G. The decreased protein level of BCAT1 in the BCAT1 knockdown HepG2 cells. (F-G) Quantitative analysis for Figure 2A-B. BCAT1, p62 and LC3-I/II protein levels in the Hela cells treated with cisplatin (20 μM) alone or plus with 20 μM CQ or 2 mM 3-MA for 24 h. (H-I) Quantitative analysis for Figure 2C-D. BCAT1, p62 and LC3-I/II protein levels in the HepG2 cells treated with cisplatin (10 μM) alone or combinated with 20 μM CQ or 2 mM 3-MA for 24 h. (J) Quantitative analysis for Figure 3A. BCAT1, p62 and LC3-I/II protein levels in Hela cells overexpressing BCAT1. (K) Quantitative analysis for Figure 3B. BCAT1, p62 and LC3-I/II protein levels in BCAT1 knockdown HepG2 cells. (L) Quantitative analysis for Figure 3C. BCAT1, mTOR, p70S6K

and 4E-BP1 and their phosphorylated counterparts expression in Hela cells overexpressing BCAT1. (M) Quantitative analysis for Figure 3D. BCAT1, mTOR, p70S6K, 4E-BP1 and their phosphorylated counterparts expression in BCAT1 knockdown HepG2 cells. All data were obtained from three independent experiments. ns, not significant. *$P < 0.05$, **$P < 0.01$, ***$P < 0.001$.

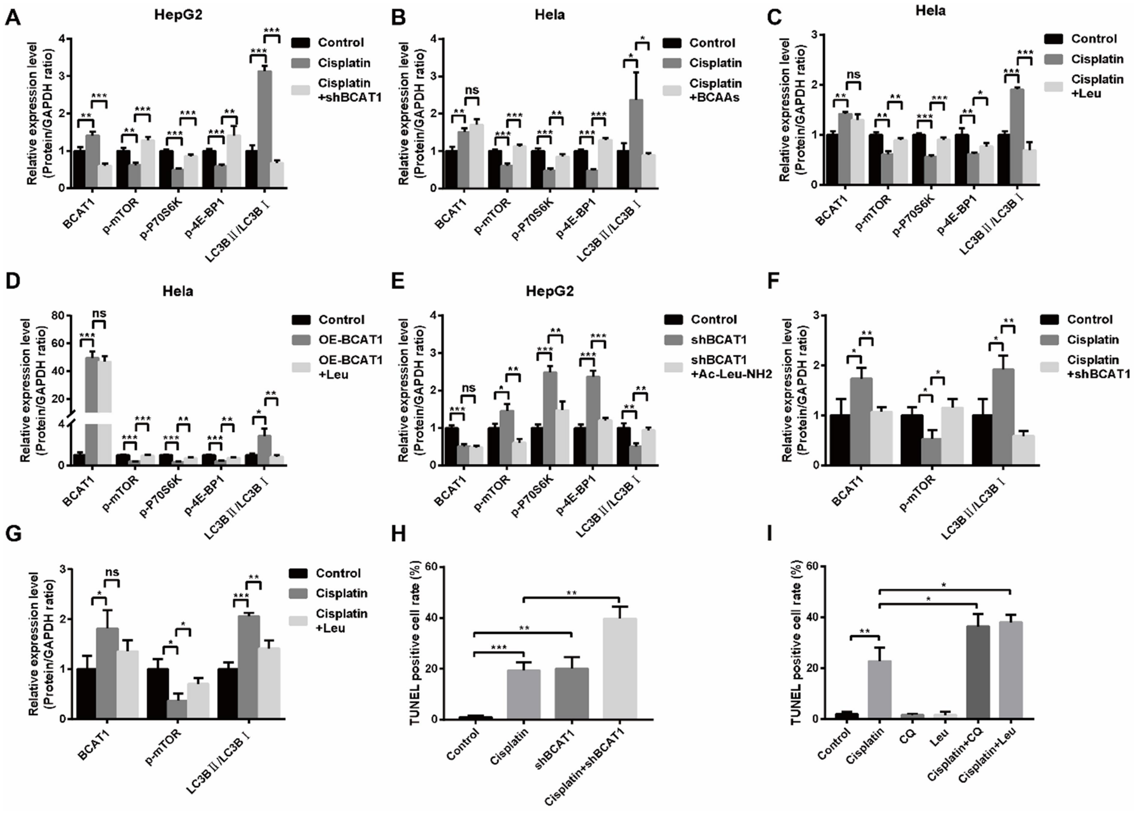

Figure S9. Qualification and statistics for the results of Western blot and TUNEL staining. (A) Quantitative analysis for Figure 3E. BCAT1, mTOR, p70S6K, 4E-BP1 and their phosphorylated counterparts and LC3-I/II expression in HepG2 or BCAT1 knockdown HepG2 cells treated with cisplatin (10 μM). (B-C) Quantitative analysis for Figure 4A-B. BCAT1, mTOR, p70S6K, 4E-BP1 and their phosphorylated counterparts and LC3-I/II expression in Hela cells treated with cisplatin (20 μM) alone or in combination with BCAAs (5 mM) or Leu (2 mM) for 24 h. (D) Quantitative analysis for Figure 5A. BCAT1, mTOR, p70S6K, 4E-BP1 and their phosphorylated counterparts and LC3-I/II expression in Hela cells with BCAT1 overexpression or in combination with Leu (2 mM) treatment for 24 h. (E) Quantitative analysis for Figure 5B. BCAT1, mTOR, p70S6K, 4E-BP1 and their phosphorylated counterparts and LC3-I/II expression in HepG2 cells with BCAT1 knockdown or in combination with an Ac-Leu-NH2 (5 mM) treatment for 24 h. (F-G)

Quantitative analysis for Figure 6E and 7H. BCAT1, LC3B-I/II, mTOR and phosphorylated mTOR expression in the tumor tissues. (H-I) Quantitative analysis for TUNEL staining in Figure 6F and 7I. All data were obtained from three independent experiments. ns, not significant. $^{*}P < 0.05$, $^{**}P < 0.01$, $^{***}P < 0.001$.
